# Long-Term Care Service Use and Caregiver Burden, Depression, and Health: A Systematic Review and Meta-Analysis

**DOI:** 10.1093/geroni/igab046.940

**Published:** 2021-12-17

**Authors:** Wayne Chong, Cindy Ng, Ringo Ho

**Affiliations:** 1 GeroPsych Consultants Pte Ltd, Singapore, Singapore; 3 Nanyang Technological University, Singapore, Singapore, Singapore

## Abstract

This study examined whether long-term care service use (LTCSU) is associated with informal caregivers’ burden, depression, and health status. Eligible articles collected data directly from caregivers, written in English, and allowed for extraction or computation of effect sizes. MEDLINE, PsycINFO and ProQuest Dissertations & Theses Global databases were searched between September 2017 and January 2018. The risk of bias of individual studies was assessed regarding confounding, study power, and other biases. This unfunded study was registered with PROSPERO: CRD42018108827. Of the 419, 209 and 346 articles identified, 24, 14 and 15 articles that involved 12,530, 6,687 and 7,331 informal caregivers respectively, were eligible for analyses regarding the above associations. With unadjusted effect sizes, omnibus tests found statistically non-significant overall effect estimates in the association of LTCSU with caregiver burden, depression, and health status. Subgroup analyses, however, revealed that the above associations differed by service type, caregiver sex, and country, respectively.

